# Evaluation of an inflammation-based score for identification of appropriate patients for comprehensive genomic profiling

**DOI:** 10.1007/s12672-022-00574-2

**Published:** 2022-10-19

**Authors:** Naomi Hayashi, Ippei Fukada, Akihiro Ohmoto, Masumi Yamazaki, Xiaofei Wang, Mari Hosonaga, Shunji Takahashi

**Affiliations:** 1 Department of Genomic Medicine, The Cancer Institute Hospital of Japanese Foundation for Cancer Research, 3-8-31 Ariake, Koto, Tokyo 135-8550 Japan; 2 Medical Oncology, The Cancer Institute Hospital of Japanese Foundation for Cancer Research, Tokyo, Japan; 3 Breast Medical Oncology, The Cancer Institute Hospital of Japanese Foundation for Cancer Research, Tokyo, Japan; 4 The Center for Advanced Medical Development, The Cancer Institute Hospital of Japanese Foundation for Cancer Research, Tokyo, Japan

**Keywords:** mGPS, Comprehensive genomic profiling, Prognosis

## Abstract

Performance status (PS) is widely used as an assessment of general condition in patients before performing comprehensive genomic profiling (CGP). However, PS scoring is dependent on each physician, and there is no objective and universal indicator to identify appropriate patients for CGP. Overall, 263 patients were scored using the modified Glasgow prognostic score (mGPS) from 0 to 2 based on the combination of serum albumin and c-reactive protein (CRP): 0, albumin ≥ 3.5 g/dl and CRP ≤ 0.5 mg/dl; 1, albumin < 3.5 g/dl or CRP > 0.5 mg/dl; and 2, albumin < 3.5 g/dl and CRP > 0.5 mg/dl. Overall survival was compared between mGPS 0–1 and mGPS 2 groups. The prognosis of patients with PS 0–1 and mGPS 2 was also evaluated. Thirty-nine patients (14.8%) were mGPS 2. Patients with mGPS 2 had significant shorter survival (14.7 months vs 4.6 months, p < 0.01). Twenty-eight patients were PS 0–1 and mGPS 2, and their survival was also short (5.6 months). Evaluation of mGPS is a simple and useful method for identifying patients with adequate prognosis using CGP.

## Introduction

Next-generation sequencing (NGS) is a remarkable technology for detecting genomic alterations in DNA and RNA sequences [1], and enables the high burden of genomic analysis to be undertaken in a single sequence run at low cost [2]. As technology advances, comprehensive genomic profiling (CGP) has been widely used in oncology practice. The primary goal of patients undergoing CGP is to have rapid access to molecular targeted therapy. Patients must participate in clinical trials to gain access to these drugs because it is often not covered by insurance. However, drug accessibility in clinical practice is very low [3–8]. The most common reason for not receiving targeted therapy was that by the time the CGP results were explained to the patients, their performance status (PS) had already declined and they were considered to have a poor prognosis [6]. One of the reasons leading to this situation is the use of PS for the assessment of general condition at the time of performing CGP. PS is a powerful prognostic marker and is used by many physicians to identify appropriate patients for performing CGP in cancer care. However, PS is dependent on the individual physician’s judgement. Neeman et al. reported that physicians' PS ratings do not seem to predict important outcomes [9]. Additionally, blind enforcement of CGP has a negative impact on the healthcare economy [2]. Hence, objective indicators are needed when implementing CGP.

Inflammation-based scores, such as GPS or modified GPS (mGPS), have been reported as good predictive indicators in cancer patients. GPS is a complex indicator that uses C-reactive protein (CRP) and serum albumin. CRP is an acute phase protein whose expression is increased by interleukin-6 (IL-6) activity [10]. IL-6 represents the degree of inflammation in cancer tissue, and IL-6 elevation leads to increased CRP and decreased serum albumin. Thus, GPS is indirect indicator of cancer cachexia and an independent prognostic marker regardless of disease stage [11]. Because various types of cancer patients in various situations receive CGP, it is considered to be meaningful to evaluate GPS in these patients. Additionally, GPS is an objective indicator, unlike PS. Hence, we evaluated whether GPS is useful compared with PS in patients receiving CGP.

## Methods

### Patients

We retrospectively analyzed collected data from consecutive patients who underwent CGP using the FoundationOne CDx Cancer Genomic Profile (Cambridge, MA, USA) or the OncoGuide NCC oncopanel system (Tokyo, Japan) at the Cancer Institute Hospital of Japanese Foundation for Cancer Research (JFCR) between November 2019 and July 2021 [12, 13]. Patient data comprised age, sex, Eastern Cooperative Oncology Group PS (ECOG PS) [14], serum albumin, and CRP. Laboratory data were used within 2 weeks before or after CGP. In the present study, GPS or mGPS was determined according to best predictive value of serum CRP using receiver operating characteristic (ROC) curves [15]. On the basis of this analysis, the cut-off value for CRP was calculated to be 0.45 mg/dl (sensitivity 69.1%, specificity 59.8%). Hence, in this study, mGPS was used because of its closer cut-off value. mGPS was scored 0 to 2 on the basis of the combination of serum albumin and CRP: 0, albumin ≥ 3.5 g/dl and CRP ≤ 0.5 mg/dl; 1, albumin < 3.5 g/dl or CRP > 0.5 mg/dl; and 2, albumin < 3.5 g/dl and CRP > 0.5 mg/dl [15].

### Statistical analysis

We used chi-squared test and Mann–Whitney U test to compare patients with mGPS 0–1 and mGPS 2. Overall survival (OS) was estimated using the Kaplan–Meier method and the log-rank test. Data were censored on 31 December 2021. Patients who were lost to follow-up were censored at the date of last contact or follow-up. OS was calculated from the date of performing CGP to the date of death from any cause. We performed univariate and multivariate analyses to estimate factors potentially prognostic for OS by calculating hazard ratios using the Cox proportional hazards model. The level of significance was set at p < 0.05 for univariate and multivariate analyses and was two-sided. All analyses were performed using EZR (www.r-project.org) [16].

### Ethics approval and consent to participate

All procedures were performed in accordance with the ethical standards of the responsible committee on human experimentation (institutional and national) and with the Helsinki Declaration of 1964 and later versions. This observational study was approved by the institutional review board of the JFCR (approval number 2021-GA-1075). Patient consent for this study was in the form of an opt-out form.

## Results

### Patients’ characteristics

In total, 341 patients were included in this study. Of these, 78 patients were excluded because mGPS could not be scored due to a lack of laboratory data. The remaining 263 patients were enrolled in this study. The median age was 58 years (range, 18–84 years). The median observation time was 8.4 months (range, 0.1–24.7 months). At the time of censoring, 127 patients (48.2%) had died from the primary disease. The median survival time was 11.8 months (range, 0.1–22.2 months). The patients were classified into two groups according to mGPS score (mGPS 0–1 and mGPS 2). Thirty-nine patients (14.8%) were mGPS 2. The characteristics of the 263 patients are shown in Table 1. Patients with mGPS 2 were older and had worse PS. The median turnaround time was 1.4 months (range, 0.8–3.3 months). No patients underwent liquid-based CGP.

**Table 1:** Association of mGPS score with clinical characteristics

Characteristics		mGPS score 0–1 (n = 224)		mGPS score 2 (n = 39)		*P* value
Age	Median (Range)	56 (13–84)		64 (35–81)		0.01^a^
PS	0–1	207	(92.4)	28	(71.8)	< 0.01^a^
	2–3	17	(7.6)	11	(28.2)	
Sex	Male	92	(41.1)	21	(53.8)	0.28
	Female	132	(58.9)	18	(46.2)	
Primary site	Colon	38	(17.0)	4	(10.3)	0.35
	Pancreas	35	(15.6)	10	(25.6)	0.16
	Breast	23	(10.3)	5	(12.8)	0.58
	Ovarian	20	(8.9)	2	(5.1)	0.75
	Sarcoma	20	(8.9)	0	(0.0)	NA
	Biliary	14	(6.2)	5	(12.8)	0.17
	Stomach	13	(5.8)	1	(2.6)	0.70
	Head and neck	8	(3.6)	1	(2.6)	1.00
	Endometrioid	7	(3.1)	1	(2.6)	1.00
	Lung	7	(3.1)	2	(5.1)	0.62
	Cervix	6	(2.7)	1	(2.6)	1.00
	Thyroid	5	(2.2)	1	(2.6)	1.00
	CUP	5	(2.2)	0	(0.0)	NA
	Esophagus	3	(1.3)	3	(7.7)	0.04^a^
	Urothelial	3	(1.3)	1	(2.6)	0.47
	Melanoma	3	(1.3)	0	(0.0)	NA
	Prostate	2	(0.9)	0	(0.0)	NA
	Hepatocellular	1	(0.4)	0	(0.0)	NA
	Duodenum	1	(0.4)	0	(0.0)	NA
	Kidney	0	(0.0)	1	(2.6)	NA
	Others	10	(4.5)	1	(2.6)	1.00
Type of CGP	Tissue	224	(100.0)	38	(100.0)	NA
	Liquid	0	(0.0)	0	(0.0)	NA

*mGPS* modified Glasgow prognostic score, *PS* performance status, *CGP* comprehensive genomic profiling, *CUP* cancer of unknown primary

^a^significance value

### Short-term survival

Thirteen patients (4.9%) had already died before the CGP results were explained. Nine of the 13 patients were mGPS 2. It is noteworthy that approximately a quarter of patients in the mGPS 2 group had already died.

### Long-term survival

**Fig. 1:**
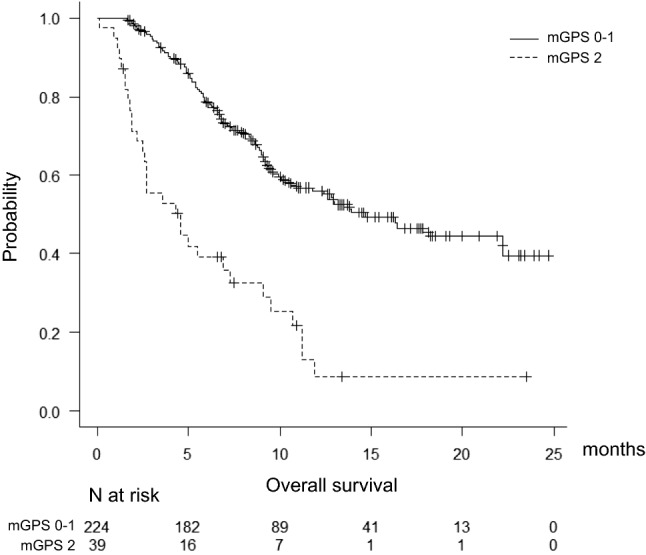
Kaplan–Meier curves for overall survival (OS). There was a significant difference between the mGPS 0–1 and mGPS 2 groups

Figure 1 shows overall survival. Patients in the mGPS 2 group had significantly shorter survival (14.7 months vs 4.6 months, p < 0.01).

### Risk factor of prognosis

Univariate and multivariate Cox proportional hazard analyses indicated that PS2–3 and mGPS 2 were significant poor prognostic factors (Table 2). PS and mGPS did not have multicollinearity (VIF: 1.0).

**Table2:** Univariate and multivariate analysis of prognosis

	Univariate			Multivariate		
	HR	95%CI	*P* value	HR	95%CI	*P* value
Age ≥ 65	1.28	0.90–1.82	0.17	0.67	0.45–0.99	0.05
Male	0.99	0.68–1.43	0.94	1.29	0.91–1.84	0.16
PS 2	3.68	2.99–5.92	< 0.01	3.14	1.92–5.15	< 0.01^a^
mGPS 2	3.65	2.41–5.39	< 0.01	3.61	2.32–5.61	< 0.01^a^

*PS* performance status, *mGPS* modified glasgow prognostic score, *HR* hazard ratio, *CI* confidence interval

^a^significance value

### Correlation between PS and mGPS

**Fig. 2:**
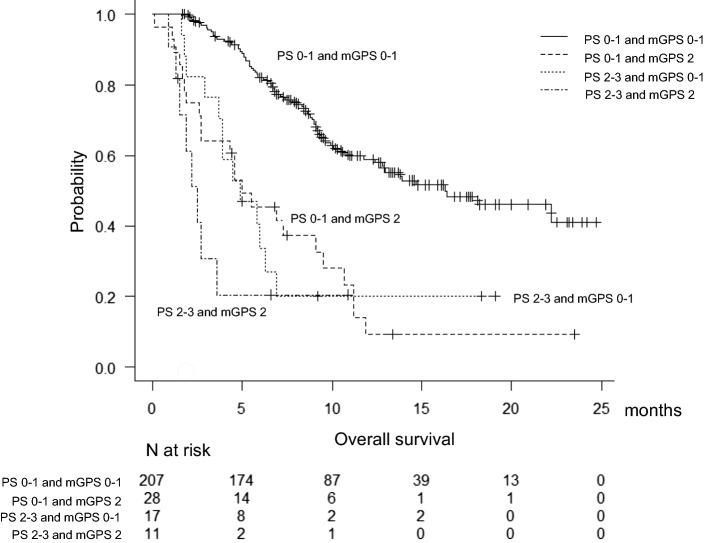
Kaplan–Meier curves for overall survival (OS) according to four categories. Patients with PS 0–1 and mGPS 2 had short survival, similar to patients with PS2–3 and mGPS 2

Two hundred thirty-five patients were PS 0–1. Of those, 28 patients (11.9%) were mGPS 2. Therefore, we classified patients into four groups to evaluate the utility of mGPS: a) PS 0–1 and mGPS 0–1; b) PS 0–1 and mGPS 2; c) PS 2–3 and mGPS 0–1; and d) PS 2–3 and mGPS 2. Figure 2 shows the overall survival of these four groups. Only patients with group a had good survival (median, 16.3 months). Other 3 groups had poor survival (median, b; 5.0 months, c; 4.9 months and d; 2.5 months). There was significant difference between group a and group c (p < 0.01). Particularly noteworthy is that group b had short survival, similar to group d (p = 0.16).

## Discussion

This is the first study to evaluate overall survival after CGP using mGPS. Our study showed that patients with mGPS 2 had a poor prognosis. More than 10% of patients were judged as PS 0–1 by their physicians, but they were in fact mGPS 2. Notably, these patients’ prognoses were very short. These results indicate that PS is insufficient to identify appropriate patients with adequate prognosis for performing CGP. Physicians tend to assign a good PS score to their patients. According to Neeman et al., physicians frequently encountered their patients during their treatment span, and their assessment of patients may have been affected by their long-term familiarity [9]. In fact, it has been reported that the longer the relationship with the patient, the less accurate the prognostic prediction [17]. Another explanation is that physicians are hesitant to assign a low PS to a patient because the PS directly affects the treatment plan. Patients also attempt to hide their symptoms, such as chemotherapy adverse events, for fear of not being able to receive treatment. Patients who are undergoing CGP may have a long-term relationship with their physician. Hence, this is why mGPS is useful and is a good predictor of prognosis because of its objective nature.

There are several combined systematic inflammation and nutritional biomarkers associated with host–tumor interactions. GPS (mGPS) is a widely used classification that was proposed by McMillan and developed by Toyama[11, 15]. The amount of IL-6 in the circulating blood reflects the cancer state and when IL-6 is elevated, CRP is increased and albumin is decreased. Thus, it is logical that GPS is a surrogate of prognosis. Many reports focus on the relationship between GPS (mGPS) and prognosis [18–23]. Recently, the utility of the CRP–albumin ratio (CAR) has been reported [24–26]. Neutrophil–lymphocyte ratio (NLR) and platelet–lymphocyte ratio (PLR) are classical nutritional assessments using blood cell components [27]. Low nutritional status lowers lymphocyte counts and the systemic inflammatory response increases neutrophil and platelet counts. Many reports have investigated the relationship between NLR or PLR and prognosis [28–32]. However, the cut-off value is not fixed. Prognostic nutritional index (PNI) contains several indexes, among which Onodera’s PNI is widely used [33]. Some studies that have investigated the relationship between PNI and prognosis [34, 35] have reported that the cut-off value is not fixed. Thus, there are several biomarkers, but only GPS (mGPS) has a cut-off value. It is considered important for eliminating differences between physicians or facilities. Hence, GPS (mGPS) was used as an indicator in this study.

The turnaround time (TAT) of this study was 1.4 months. Considering the TAT, the prognosis of patients with GPS 2 is too short to enable participation in clinical trials. CGP should be considered before mGPS scores worsen. Several reports have investigated the advantages and disadvantages of CGP. An advantage is its potential to improve prognosis by leading to targeted therapy [36]. Targeted therapies have been reported to be cost-effective compared with standard care because CGP has the potential to reduce the use of inappropriate drugs [37–39]. Conversely, a disadvantage is its low contribution to treatment plans despite its high cost [40]. It is important to confer a benefit to patients. In any case, it would be best to conduct CGP at a time that would benefit the patient.

This study had several limitations. First, this was a single-institution retrospective study with potential selection bias and a short follow-up time. Second, treatment after CGP was not taken into consideration. Patients in the mGPS 0–1 group may have a better prognosis because of the continued treatment or because they are likely to have access to targeted therapies. Third, this cohort included subgroup which the subjective PS was better than mGPS in predicting survival. PS and mGPS were independent prognostic factors in this analysis. It is unclear whether PS or mGPS is a better indicator, however, mGPS would be at least meaningful complement when physicians assign a good PS.

In future work, it would be important to extend this analysis to show the relationship between the PS/mGPS combination and the CGP results since identification of pro-inflammatory mutations is of considerable interest to the scientific and medical community.

## Conclusion

Our study demonstrated that evaluation of mGPS may help physicians to identify appropriate patients with an adequate prognosis to receive CGP.

## Data Availability

The datasets generated during and/or analyzed during the current study are available from the corresponding author on reasonable request.
